# Using RegGAN to generate synthetic CT images from CBCT images acquired with different linear accelerators

**DOI:** 10.1186/s12885-023-11274-7

**Published:** 2023-09-05

**Authors:** Zhenkai Li, Qingxian Zhang, Haodong Li, Lingke Kong, Huadong Wang, Benzhe Liang, Mingming Chen, Xiaohang Qin, Yong Yin, Zhenjiang Li

**Affiliations:** 1 Chengdu University of Technology, Chengdu, China; 2 Department of Radiation Oncology Physics and Technology, Shandong Cancer Hospital and Institute, Shandong First Medical University and Shandong Academy of Medical Sciences, Jinan, China; 3 Manteia Technologies Co., Ltd., Xiamen, China

**Keywords:** Radiotherapy, Deep learning, CBCT, sCT, RegGAN

## Abstract

**Background:**

The goal was to investigate the feasibility of the registration generative adversarial network (RegGAN) model in image conversion for performing adaptive radiation therapy on the head and neck and its stability under different cone beam computed tomography (CBCT) models.

**Methods:**

A total of 100 CBCT and CT images of patients diagnosed with head and neck tumors were utilized for the training phase, whereas the testing phase involved 40 distinct patients obtained from four different linear accelerators. The RegGAN model was trained and tested to evaluate its performance. The generated synthetic CT (sCT) image quality was compared to that of planning CT (pCT) images by employing metrics such as the mean absolute error (MAE), peak signal-to-noise ratio (PSNR), and structural similarity index measure (SSIM). Moreover, the radiation therapy plan was uniformly applied to both the sCT and pCT images to analyze the planning target volume (PTV) dose statistics and calculate the dose difference rate, reinforcing the model’s accuracy.

**Results:**

The generated sCT images had good image quality, and no significant differences were observed among the different CBCT modes. The conversion effect achieved for Synergy was the best, and the MAE decreased from 231.3 ± 55.48 to 45.63 ± 10.78; the PSNR increased from 19.40 ± 1.46 to 26.75 ± 1.32; the SSIM increased from 0.82 ± 0.02 to 0.85 ± 0.04. The quality improvement effect achieved for sCT image synthesis based on RegGAN was obvious, and no significant sCT synthesis differences were observed among different accelerators.

**Conclusion:**

The sCT images generated by the RegGAN model had high image quality, and the RegGAN model exhibited a strong generalization ability across different accelerators, enabling its outputs to be used as reference images for performing adaptive radiation therapy on the head and neck.

## Introduction

The goal of radiotherapy is to maximize the dose applied to the target tumor while minimizing the dose affecting the surrounding organs at risk. However, due to anatomical changes that may occur during the typical multiweek treatment period, failure to modify the radiotherapy plan in a timely manner according to these changes can result in imprecise dose delivery, compromising treatment efficacy and even causing radiation-induced reactions in normal tissues. Adaptive radiotherapy is a promising solution that can adjust treatment plans in a timely manner based on daily patient images. However, it still has drawbacks such as its long processing time and lack of automated assisting tools [1]. Cone beam computed tomography (CBCT) is a commonly used tool for observing tumor changes, but its image quality is poor, and its Hounsfield unit (HU) values are inaccurate due to the effects of scattering, making CBCT unsuitable for developing radiotherapy plans [2–5]. Therefore, many studies [6–12] have attempted to achieve improved CBCT image quality and HU accuracy by using traditional physical modeling methods to correct X-ray scattering results, but due to their long processing times and other limitations, these methods have not yet been widely used in clinical applications.

The main type of head and neck tumor is squamous cell carcinoma, which responds well to radiotherapy [13]. However, due to the concentration of critical organs and the high likelihood of anatomical changes in head and neck during tumor radiotherapy, precise dose delivery is crucial for minimizing the dose applied to normal tissues. Failure to modify the original radiotherapy plan based on anatomical changes can lead to insufficient tumor tissue doses and excessive doses applied to critical organs, ultimately affecting clinical treatment outcomes [13–15].

As an important image guidance method in adaptive radiotherapy, CBCT cannot be directly used for treatment due to dose calculation errors caused by scattering and other factors. Converting CBCT images to high-quality synthetic CT (sCT) images improves the accuracy of patient-adaptive radiotherapy and reduces the dose applied to organs at risk (OARs). Recently, many studies have been conducted on the use of deep learning models for sCT image generation in adaptive radiotherapy.

CBCT-guided adaptive radiotherapy is still the main trend. Recently, many studies have combined deep learning methods with adaptive radiotherapy. Several models have been proposed for image-to-image translation, among which the most common methods are U-Net [16–19] and generative adversarial networks (GANs) [20–25]. U-Net utilizes global and local features in the spatial domain to suppress scatter artifacts for matching tasks. In contrast, the GAN architecture employs a generator and a discriminator for adversarial competition, and the total loss of both modules is computed to make the generated sCT images more realistic. Networks based on the GAN architecture are superior to other methods.

Several studies have applied the registration GAN (RegGAN) model to generate sCT images for adaptive radiotherapy. Wang et al. [23] utilized the RegGAN model to improve the quality of daily CBCT images and the accuracy of HU values in adaptive radiotherapy for esophageal cancer and used the synthesized sCT images for dose calculation purposes during radiotherapy. The results demonstrated a significant improvement in the quality of the sCT images generated by RegGAN over that of the original CBCT images, with higher dose calculation accuracy during a gamma analysis. Suwanraksa et al. [26] applied the RegNet model to the treatment of head and neck tumors and compared the performance of GANs trained with and without RegNet. The results showed that adding a GAN improved the network’s performance.

However, limited research has been conducted on verifying a model’s universality by applying it to different accelerators. This study aims to use RegGAN to improve the quality of CBCT images acquired from different accelerators and the accuracy of the associated HU values. The synthesized pseudo-CT images are then used for radiotherapy dose calculation, and their accuracy is compared with that of planning CT (pCT) doses to verify the dose calculation precision of the proposed approach. This study provides a reference for image conversion in adaptive radiotherapy.

## Materials and methods

### Clinical dataset

CT and CBCT images acquired from 100 patients with head and neck tumors treated on a Varian Vital Beam accelerator (Varian Medical System, USA) at the Shandong Cancer Hospital were included in this study. Eighty cases were used as the training set, and 20 cases were used as the validation set. In addition, CT and CBCT images from 40 patients with head and neck tumors (10 from each accelerator) treated on four accelerators - Halcyon (Varian Medical System, USA), Trilogy (Varian Medical System, USA), Varian Vital Beam (Varian Medical System, USA), and Synergy (Elekta Corporation, Sweden) - were used as the test set. All patients’ pCT images were obtained using the Brilliance big-bore CT positioning machine (Philips, Amsterdam, Netherlands), with each patient lying in the supine position and their head and neck region secured using a vacuum bag and thermoplastic mask. The scanning layer thickness was 3 mm. The pCT and CBCT scanning parameters are shown in Table 1.

**Table 1:** Scanning and reconstruction parameters of CBCT and pCT

	Tube voltage (kVP)	Tube current (mA)	Spatial resolution (mm^2^)	Slice thickness (mm)	Image size	Number of slices
CT	120	260	0.977 × 0.977	3 mm	512 × 512	42 ~ 65
Halcyon	100	30	0.55 × 0.55	3 mm	512 × 512	63
Trilogy	100	10	0.651 × 0.651	3 mm	384 × 384	70
Vital beam	100	20	0.625 × 0.625	3 mm	512 × 512	54
Synergy	120	40	1 × 1	2.5 mm	410 × 410	264

### Image Preprocessing

Since the pCT and CBCT images were acquired at different time points and under different field of view (FOV) conditions, it was essential to ensure the accuracy of the image comparison. We used the MIM 7.1.9 workstation (MIM Software Inc., USA) for the rigid registration of the pCT and CBCT images, with the pCT images as the primary sequence and the CBCT images as the secondary sequence. The images were then cropped to the same number of slices. To prevent high-density bone structures with elevated HU values from impacting the training process, the HU values of both image types were limited to a range of [-1000, 2000] and normalized to [-1, 1].

### RegGAN Model

The RegGAN model is composed of three main parts: a generator network, a registration network, and a discriminator network. The generator network is responsible for generating synthesized images, the registration network is responsible for correcting label noise, and the discriminator network aims to distinguish between real images and generated images.

The generator network is composed of a ResNet-like structure, which includes 2 downsampling convolution blocks with a 3 × 3 kernel and a 2 × 2 stride, 9 residual blocks, and 2 upsampling deconvolution blocks with a 3 × 3 kernel and a 2 × 2 stride. The registration network employs U-Net due to its capability to extract both global and local features in the spatial domain, effectively mitigating global scattering artifacts and local artifacts. The discriminator is designed with four layers of 4 × 4 convolutional kernels and a stride of 4 for full convolution. Convolution is employed to map the input to an N×N matrix, where each point represents an evaluation value for a small region within the original image. Finally, the output determines the authenticity of the image by assigning either a 0 (real) or a 1 (fake).

**Fig. 1:**
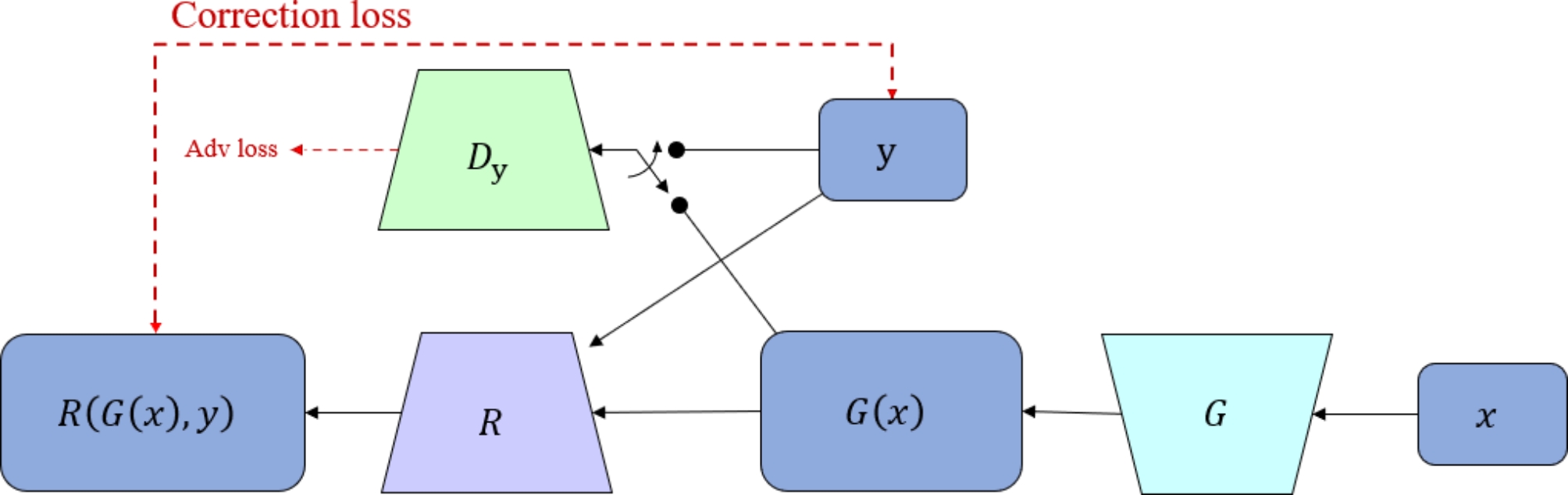
RegGAN model network structure

**Fig. 2:**
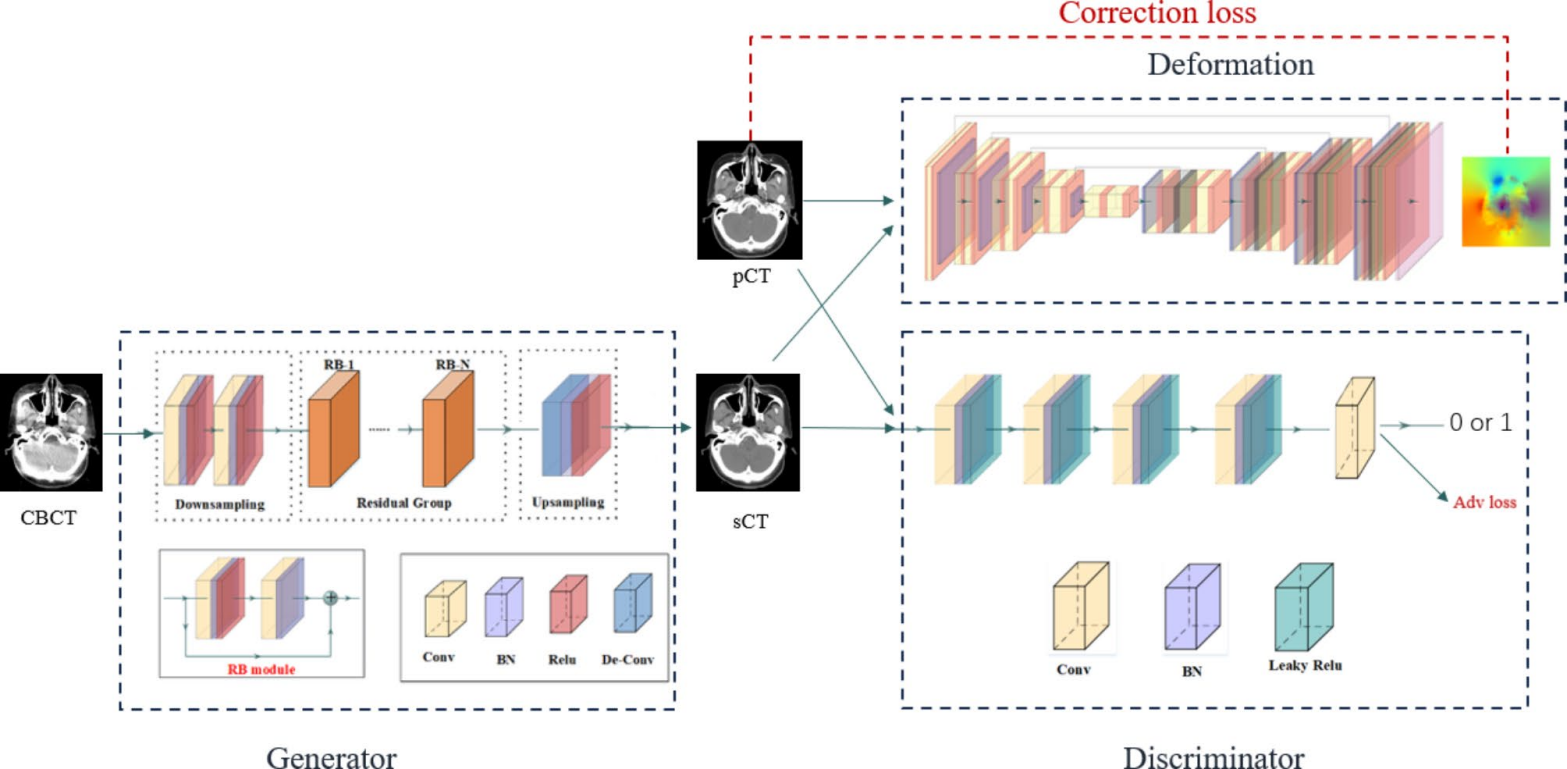
RegGAN model details

In the model, unaligned images are considered noisy images, and the image transformation training process of the model is converted into an unsupervised learning process with noisy labels. Given a training set with N noisy labels $$ {\{({x}_{n} , {\stackrel{\sim}{y}}_{n})\}}_{n=1}^{N}$$, where $$ {x}_{n}, {\stackrel{\sim}{y}}_{n}$$ are images with two different modalities, we assume that $$ {x}_{n}$$ and $$ {y}_{n}$$ are the correctly aligned label images, but in reality, they are unknown. Utilizing the generator $$ G$$, as shown in Eq. (1), on $$ {\{({x}_{n} , {\stackrel{\sim}{y}}_{n})\}}_{n=1}^{N}$$ is as equivalent as possible to the noise-free dataset $$ {\{({x}_{n} , {y}_{n})\}}_{n=1}^{N}$$. The model structure is shown in Figs. 1 and 2.

1 $$\hat  {G}=\underset{G}{argmin}\frac{1}{N}{\sum }_{n=1}^{N}L(\varphi \circ G({x}_{n}),{\stackrel{\sim}{y}}_{n})$$

The model aims to correct the output of the generator $$ G\left({x}_{n}\right)$$ by modeling the noise transition to match the noise distribution. Since the type of noise distribution is relatively certain, it can be represented as a displacement error $$ \stackrel{\sim}{y}=y\circ T$$, where $$ T$$ denotes a random deformation field that causes random displacement for each pixel. Therefore, a registration network $$ R$$ is used after the generator $$ G$$ as a label noise model to correct the results. The correction loss equation is shown in Eq. (2):

2 $$ \underset{G,R}{min}{\mathcal{L}}_{\text{Corr }}(G,R)={\mathbb{E}}_{x,\stackrel{\sim}{y}}[\parallel \stackrel{\sim}{y}-G(x)\circ R(G\left(x\right),\stackrel{\sim}{y}\left){\parallel }_{1}\right]$$

where $R\left(G\left(x\right),\stackrel{\sim}{y}\right)$ represents the deformation field, and $\circ$ denotes the resampling operation. To evaluate the smoothness of the deformation field and minimize the deformation field gradient, a smoothness loss is calculated using Eq. (3).

3 $$ \underset{R}{min}{\mathcal{L}}_{\text{Smooth }}\left(R\right)={\mathbb{E}}_{x,\stackrel{\sim}{y}}[\parallel \nabla R(G\left(x\right),\stackrel{\sim}{y}\left){\parallel }^{2}\right]$$

Finally, the correction loss $$ {\mathcal{L}}_{\text{Corr }}$$, smoothness loss $$ {\mathcal{L}}_{\text{smooth }}$$, and adversarial loss $$ {\mathcal{L}}_{\text{Adv}}$$ between the generator and discriminator $$ D$$ form the final total loss equation, as shown in Eq. (4).

4 $$ \underset{G,R}{min}\underset{D}{max}{\mathcal{L}}_{\text{Total }}\left(G,R,D\right)={\mathcal{L}}_{\text{Corr }}+{\mathcal{L}}_{\text{Smooth }}+{\mathcal{L}}_{\text{Adv}}$$

### Training

All training procedures were conducted on a 64-bit Ubuntu Linux system using PyTorch software, with a system configuration containing 96 GB of RAM and a 24-GB Nvidia Titan RTX GPU. All images were normalized to the range of [-1, 1] and resampled to a size of 256 × 256. The adaptive moment estimation (Adam) optimizer was used for training with a learning rate of 1e-4 and (β1, β2) = (0.5, 0.999). The batch size was set to 1 with a weight decay of 1e-4. The training process included 80 epochs, covering a total of 640k iterations. The training duration was estimated to be approximately 7 h, while the average image processing time required per patient was approximately 15 s.

### Image evaluation

In the image quality assessment, pCT was used as the ‘gold standard’ for evaluating both the CBCT and sCT images. The image quality was evaluated using mean absolute error (MAE), peak signal-to-noise ratio (PSNR), and structural similarity index measure (SSIM) metrics. A smaller MAE value, along with larger PSNR and SSIM values, indicates higher similarity between the two tested images.

5 $$ MAE=\frac{\sum _{1}^{x}\sum _{1}^{y}{\sum }_{1}^{z}|sC{T}_{xyz}-pC{T}_{xyz}|}{xyz}$$

6 $$ MSE={\frac{\sum _{1}^{x}\sum _{1}^{y}{\sum }_{1}^{z}|sC{T}_{xyz}-pC{T}_{xyz}|}{xyz}}^{2}$$

7 $$ PSNR=10{log}(\frac{{max}_{CT}^{2}}{MSE})$$

8 $$ SSIM=\frac{\left(2{\mu }_{sCT}{\mu }_{pCT}+{c}_{1}\right)\left(2{\sigma }_{sCT,pCT}+{c}_{2}\right)}{\left({\mu }_{sCT}^{2}+{\mu }_{pCT}^{2}+{c}_{1}\right)\left({\sigma }_{sCT}^{2}+{\sigma }_{pCT}^{2}+{c}_{2}\right)} $$

$$ x,y,z$$ correspond to the values of the coordinates (x, y, z) within an image, and $$ xyz$$ denotes the total number of voxels that are present in the image. $$ {max}_{CT} $$is the maximum pixel value of the pCT or sCT images; MSE is the mean squared error; $$ {\mu }_{sCT}$$ and $$ {\mu }_{pCT}$$ are the means of the sCT and pCT images, respectively; $$ {\sigma }_{sCT,pCT}$$ denotes the standard deviations of the images; $$ {\sigma }_{sCT,pCT}$$ is the cross-covariance; c1 and c2 are luminance and contrast regularization constants, respectively.

### Dose evaluation

Radiation therapy plans were designed using both pCT and sCT images with consistent physical planning conditions. The differences among the $$ {D}_{min}$$, $$ {D}_{max}$$, $$ {D}_{mean}$$, $$ {D}_{2}$$, $$ {D}_{50}$$, $$ {D}_{95}$$ and $$ {D}_{98}$$ values within the PTV were compared to evaluate the accuracy of the sCT dose calculation results. The difference rate (Dr) was calculated using the formula shown in Eq. (9):

5 $$ Dr=\frac{{D}_{sCT}-{D}_{pCT}}{{D}_{pCT}} $$

where $$ {D}_{sCT}$$ represents the dose values calculated on the generated images, and $$ {D}_{pCT}$$ represents the dose values calculated according to the original plan.

### Statistical analysis

A statistical analysis was performed using SPSS 20.0. The data were represented as (x̅±s). Nonparametric tests were used to compare the sCT image values and dose difference rates across different accelerators. Finally, pairwise comparisons were performed using the Kruskal‒Wallis corrected P value. The level of statistical significance was set at p < 0.05.

## Results

### Image quality comparison

The image quality of the sCT images was greatly improved over that of CBCT images across all four accelerators, with the most significant improvement observed on the Synergy accelerator. The MAE decreased from 231.3 ± 55.48 to 45.63 ± 10.78; the PSNR increased from 19.40 ± 1.46 to 26.75 ± 1.32; and the SSIM increased from 0.82 ± 0.02 to 0.85 ± 0.04. The Vital Beam generated the best sCT image quality, with an MAE of 33.45 ± 5.78, a PSNR of 27.84 ± 0.98, and an SSIM of 0.93 ± 0.01. The sCT image quality differences among the images generated by different accelerators were relatively small. The image quality levels of the CBCT and sCT images produced by different accelerators are shown in Table 2. Table 3 presents a comparative analysis among several relevant studies aimed at improving sCT quality. In comparison with the study conducted by Wang [23], our research exhibited similar MAE values while displaying significant PSNR differences. These disparities arose due to the dissimilar selection of our ground-truth volume (GTV).

**Table 2:** Image quality parameters for different accelerators

	MAE	PSNR	SSIM
Halcyon CBCT	64.90 ± 15.53	25.12 ± 2.12	0.86 ± 0.02
sCT	42.99 ± 12.53	26.58 ± 2.23	0.88 ± 0.02
Trilogy CBCT	75.24 ± 13.62	25.32 ± 0.86	0.90 ± 0.02
sCT	34.81 ± 3.04	27.89 ± 0.75	0.91 ± 0.02
Vital beam CBCT	72.43 ± 15.76	25.52 ± 1.33	0.92 ± 0.02
sCT	33.45 ± 5.78	27.84 ± 0.98	0.93 ± 0.01
Synergy CBCT	231.3 ± 55.48	19.40 ± 1.46	0.82 ± 0.02
sCT	45.63 ± 10.78	26.75 ± 1.32	0.85 ± 0.04

**Table 3:** Comparison among the image quality results of various sCT studies

	GTV	MAE	PSNR	SSIM	Linac
CBCT [23]	Esophageal	80.10 ± 9.10	21.30 ± 4.20	/	Varian EDGE Linac (Varian, USA)
sCT (RegGAN) [23]		43.70 ± 4.80	27.90 ± 5.60	/	
CBCT [26]	H&N	58.16 ± 25.17	26.06 ± 2.44	0.82 ± 0.09	Varian True Beam STx LINAC (Varian, USA)
sCT (Pix2Pix + RegNet) [26]		41.62 ± 13.69	27.71 ± 2.32	0.86 ± 0.05	
sCT (CycleGAN + RegNet) [26]		41.67 ± 15.04	27.73 ± 2.20	0.86 ± 0.05	
sCT (UNIT + RegNet) [26]		41.34 ± 13.66	27.87 ± 2.09	0.86 ± 0.05	
CBCT [27]	H&N	197.72 ± 59.2	22.07 ± 2.80	0.95 ± 0.03	Varian Medical Systems (Varian, USA)
sCT (without respath) [27]		150.05 ± 48.7	23.69 ± 2.80	0.96 ± 0.02	
sCT (with respath) [27]		140.7 ± 54.80	24.44 ± 3.7	0.96 ± 0.02	

**Fig. 3:**
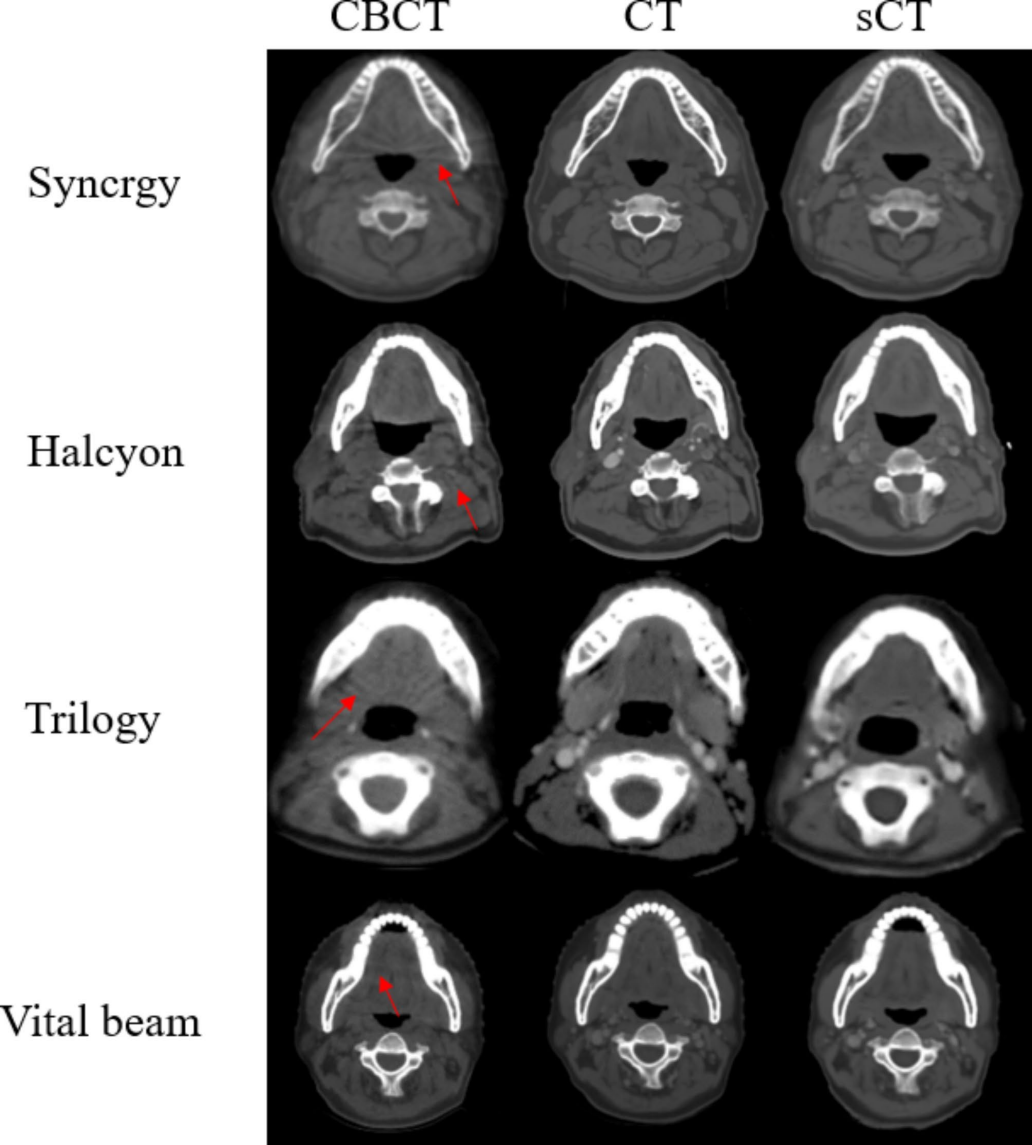
Comparison among the CBCT, CT, and sCT images for different accelerators

The red arrows in Fig. 3 indicate the air cavity artifacts, where the sCT images showed significant reductions in artifacts compared to the corresponding CBCT images. The sCT image quality differences observed across different accelerators were relatively small.

**Fig. 4:**
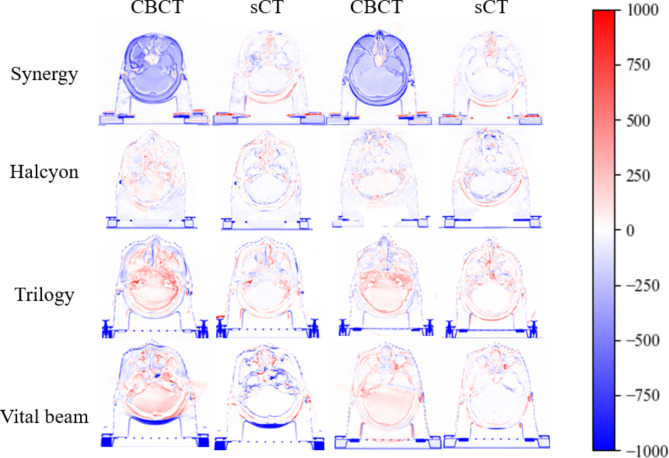
CBCT-pCT and sCT-pCT differences between different accelerators

Figure 4 shows the residual images generated from the differences between the CBCT and sCT images with pCT for 8 patients. As illustrated, the differences between the CBCT and pCT images were larger, while the differences between the sCT and pCT images were smaller, indicating a significant improvement in image quality. No apparent differences were observed among the sCT images generated by different accelerators, but the improvement effect was most evident for the Synergy accelerator.

**Fig. 5:**
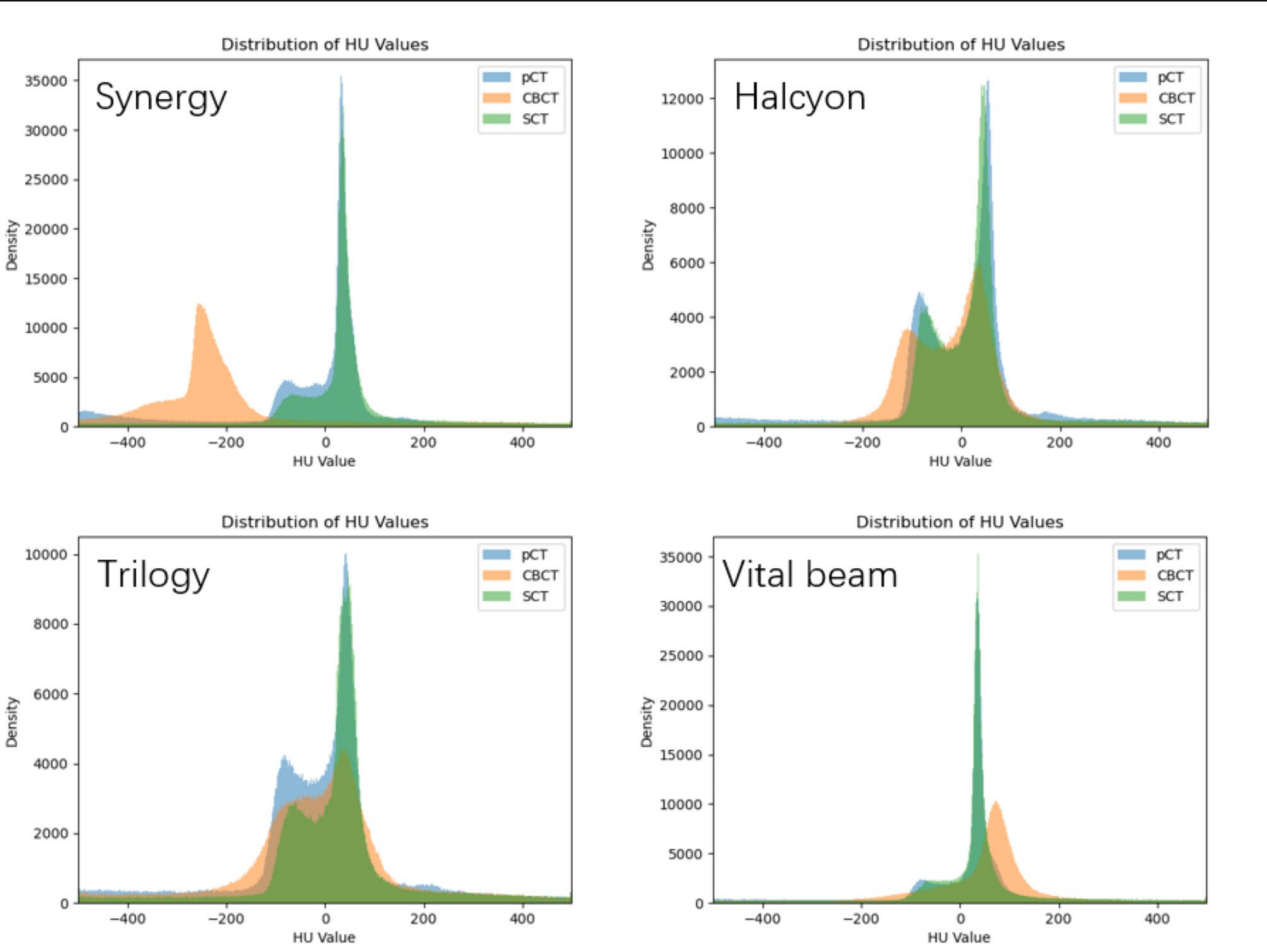
HU value differences between the CBCT, sCT, and pCT images of different accelerators

Figure 5 shows the HU value distribution curves of the patient images selected from different accelerators. Due to the preprocessing of the head and neck images, the HU values were reduced to [-1000, 2000], resulting in a very high peak near − 1000, which compressed the effective comparison range of [-500, 500] and was not conducive to observing the HU value differences. Therefore, we discarded the parts outside the [-500, 500] range during the calculation process. For Synergy, significant differences were observed between the CBCT and pCT images, with the CBCT peak at approximately − 220 and the pCT peak at approximately 70, but the sCT and pCT images exhibited a higher similarity in their HU value distributions. The CBCT images derived from other accelerators also showed significant differences from the pCT images, but the similarity between the HU value distributions of the sCT images generated by different accelerators and the pCT images was significantly improved.

### Dose calculation

**Fig. 6:**
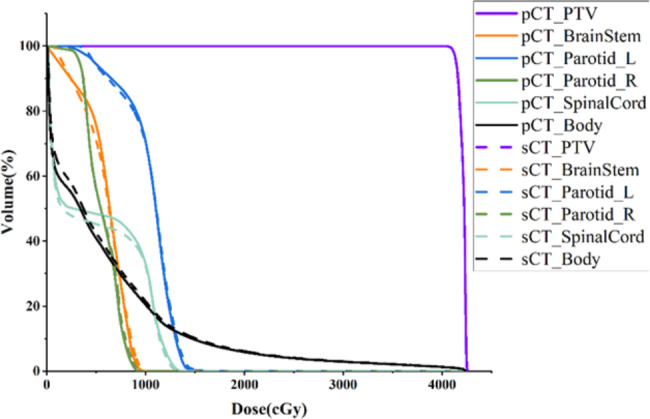
Comparison between the DVHs of pCT and sCT

The differences among the PTV doses produced by different machines are shown in Table 4. The difference in dose between the sCT and CT plans was within 3%. The dose discrepancy rates between different linacs did not show any significant differences (p < 0.05). The difference rate was relatively high and fluctuated greatly for $$ {D}_{min}$$, which may have been related to the HU value error of the sCT images. The clinical optimization goal was achieved when D95 exceeded or equaled the prescription dose. If the acceptable range for the D95 error rate in the PTV was set to 1%, then 32 out of the 40 patients in this study met the error requirements, with a pass rate of 80%. If the acceptable range was set to 3%, then the pass rate was 100%. Figure 6 displays a comparison among the results of average dose-volume histograms (DVH) produced by pCT and sCT plans for a single patient, indicating a high degree of overlap and minimal differences between the two DVH curves.

**Table 4:** Dose rate differences between the PTVs of different accelerators

	${\varvec{D}}_{\varvec{m}\varvec{i}\varvec{n}}$ (%)	${\varvec{D}}_{\varvec{m}\varvec{a}\varvec{x}}$ (%)	${\varvec{D}}_{\varvec{m}\varvec{e}\varvec{a}\varvec{n}}$ (%)	${\varvec{D}}_{2}$ (%)	${\varvec{D}}_{50}$ (%)	${\varvec{D}}_{95}$ (%)	${\varvec{D}}_{98}$ (%)
Halcyon	-0.49 ± 1.97	-0.13 ± 0.79	-0.07 ± 0.84	-0.12 ± 0.82	-0.07 ± 0.87	0.00 ± 0.85	-0.09 ± 0.81
Trilogy	0.25 ± 2.14	-0.37 ± 1.51	-0.69 ± 1.36	-0.52 ± 1.42	-0.80 ± 1.40	-0.24 ± 1.66	-0.36 ± 1.38
Vital beam	-0.34 ± 1.60	-0.04 ± 0.54	0.00 ± 0.00	0.02 ± 0.30	0.09 ± 0.20	0.10 ± 0.27	0.07 ± 0.37
Synergy	-0.03 ± 2.22	-0.72 ± 1.29	0.04 ± 0.13	-0.42 ± 1.19	-0.38 ± 1.20	-0.11 ± 1.47	-0.40 ± 2.09

## Discussion

In this study, we utilized RegGAN to convert CBCT images into pseudo-CT (sCT) images. RegGAN can perform supervised learning with noisy labels, which is similar to actual treatment scenarios in which CBCT images and pCT images are typically not acquired simultaneously. As treatment progresses, the time interval increases, and patients may experience weight loss and tumor volume reduction, resulting in significantly different CBCT and CT images. The RegGAN model accurately converts CBCT images of varying quality into precise sCT images and exhibits good universality across different accelerators. The original CBCT image quality varied among the four accelerators, and because the Synergy accelerator’s CBCT images had higher spatial resolutions than the CT images, the image quality performance index calculated with pCT as the “gold standard” was lower. However, no significant differences were observed between the quality of the generated sCT images and that of the other accelerators (p < 0.05).

The main purpose of generating sCT images during CBCT-guided adaptive radiotherapy is to create a treatment plan based on sCT. Therefore, this study also explored the possibility of using sCT images for radiation therapy dose calculations. By using pCT as the “gold standard,“ we evaluated the accuracy of the HU values produced for the generated sCT images and the accuracy of the dose calculations in actual radiation therapy plans. The dose calculation results showed no significant dose variation rate differences among the different accelerators (p < 0.05), indicating that the HU accuracy of the sCT images generated by the RegGAN network was high, and their dose distribution was similar to pCT’s “gold standard.” The differences were within the clinically acceptable range, and the image conversion speed was fast, with the ability to complete the conversion of one patient image in just 15 seconds.

## Conclusion

The RegGAN model can generate high-precision sCT images from CBCT images obtained with different accelerators. The model exhibits good generalization across different CBCT imaging models and produces dose calculations that are similar to those of pCT plans, with acceptable errors. Therefore, the RegGAN model can be used in adaptive radiotherapy for head and neck tumors.

## Data Availability

All data obtained during the current study are available from the corresponding author on reasonable request.

## Abbreviations

CBCT: Cone Beam CT
sCT: Synthetic CT
HU: Hounsfield Unit
OARs: Organs At Risk
pCT: Planning CT
FOV: Field of View
MAE: Mean Absolute Error
PSNR: Peak Signal-to-Noise Ratio
SSIM: Structural Similarity Index Measure
GTV: Gross Target Volume
PTV: Planning Target Volume
